# Round the Hospitals

**Published:** 1920-12-11

**Authors:** 


### ROUND THE HOSPITALS.

The King held the last of the War Investitures at Buckingham Palace on December 2, when His Majesty personally conferred decorations on the following nursing sisters:—Bar to the Royal Red Cross.—Miss Lilian Cushon, British Red Cross Society. The Royal Red Cross, First Class.—*Q.A.I.M.N.S.*—Miss Eva Fox, Miss Margaret Johnston, Miss Marion McCormick, Miss Margaret Smyth, Miss Winifred Teevan. *Q.A.I.M.N.S.R.*—Miss Lucy Hill, Miss Ethel Hutchings, Miss Ethel Smithies, Miss Constance Walker. *R.A.F.N.S.*—Miss Joanna Cruickshank, Miss Christina Cameron. *T.F.N.S.*—Miss Clara Battye, Mrs. Jean Bell-Edgar, Miss Zilla Hailstone,

Miss Dorothy Jones. *C.N.S.*—Miss Candace Alexander, Miss Jane Armstrong, Miss Hannah Bailie, Miss Mary Jones, Miss Charlotte Leigh. *B.R.C.S.*—Mrs. Johanna O'Malley, Miss Gertrude Stevens. *Civil and War Hospitals.*—Miss Lilian Drewitt, Miss Mercy Huffer. Queen Alexandra received the nursing sisters, together with Miss A. B. Smith, Matron-in-Chief, at Marlborough House after the Investiture. It was a very special occasion, as marking the close of the long and brilliant series of Investitures which have to some faint extent brought to the notice of the public the deeds of heroism wrought by men and women for their protection.

The Minister of Labour has given notice of his intention to give a decision on the question whether the employment by a hospital supported out of voluntary contributions of (1) trained sisters and nurses and (2) probationer nurses is such employment as to bring them under the provisions of the Unemployment Insurance Act, and especially whether such employment is employment in domestic service. This is a question of the highest importance to hospitals and the nursing profession. The decision is to be given on or after December 17, and any person or body claiming to be interested may, before this date, make written representations on the matter to the Minister, or apply to be heard orally. Such representations or applications should be addressed to the Principal Assistant Secretary, Ministry of Labour Employment Department, Queen Anne's Chambers, Westminster, S.W. 1. It should be noted that the case of private nurses working on their own or attached to a co-operation or home is apparently not to be dealt with in this decision, which is to be confined to nurses working in a hospital. We hope the necessary steps will be taken to obtain a ruling by the Minister of Labour on the position of private nurses under the Unemployment Insurance Act.

We understand that the Victory Ball on Armistice Night realised the respectable total of £7,000 when all expenses had been paid. This amount is divided between the British Red Cross and the Jubilee Institute. The habit of dancing to the tune of "National Health" is an excellent one, and we learn with pleasure that Miss May Beeman, C.B.E., has engaged the Albert Hall already for a similar occasion next year.

A successful effort, which took the form of a Sale of Work and American Tea, was made at Stoke on November 25 in aid of the North Staffordshire Infirmary and the Cripples' Hospital on the initiative of Mrs. Wheelton Hind. One of the main requirements is an expansion in the hospital, which has a waiting-list of 1,000 cases, and an extension to the Nurses' Home, which is overcrowded, as is also a temporary hut in the grounds put up for their use. The Rector of Stoke explained that the nurses were having increasingly hard work, as treatment became more complicated, and, owing to the heavy waiting-list, all the cases were acute. Unlike other people, the nurses had not had their hours reduced to any considerable extent, but the authorities wanted to reduce them to eight hours a day. To do that they must have an additional twenty-five nurses. As regards the Cripples' Hospital, the lack of accommodation for the nurses is even more deplorable. Twelve are living outside, others are housed in three huts in the garden, and one of the nurses has to sleep in a loft.

The Ladies' Auxiliary of Bristol Children's Hospital held their twenty-first annual meeting on November 26 under the presidency of the Duchess of Beaufort, who informed the meeting that their Society had now raised the magnificent sum of £20,000 for the hospital. It is associated with a flourishing Ladies' Needlework Guild, and between them they form a strong pillar of support for the institution. As the Duchess remarked, "Going from door to door is not apparently very heroic work, but the end attained is surely a victory of no mean order." This year's income to date is £1,395.

Mrs. Martin Harvey, who, with her husband, was playing in Cardiff last week, attended a meeting at the Mansion House on the Thursday, and eloquently pleaded the cause of the Nation's Fund for Nurses. The invitations issued by the Lord Mayor and Lady Mayoress had been largely responded to, and among the well-known people present, in addition to Lady Courtis, who presided, were Lady Hughes and Lady Lynn-Thomas. Mrs. Martin Harvey made a moving appeal for the Fund, saying the public did not realise how shockingly underpaid was the noblest of all professions, or it would never allow the existing state of affairs to continue. Mrs. Harvey said that during her work with the Red Cross Society on the Western Front in 1914 she saw something of the heroic work performed by British nurses, and she was looking to the Tribute Fund to alleviate the anxiety of those who had returned shattered in health and spirit and whose pensions were hopelessly inadequate. "It is not a question of charity," declared the speaker. "No nurse ever asked for that. It is a question of sacred responsibility left to us as a result of the war, and we cannot ignore it. Subscribe to the Fund in memory of someone dear who came back to you, or in memory of one who fell for liberty." Mr. Martin Harvey recited "A Hymn of Love for England," and members of his company rendered musical items.

It is announced from official sources that there are still a large number of Royal Red Crosses awaiting issue. Nursing sisters and other ladies who have not received their decoration are requested to apply in writing to the Secretary C. 2 [Investiture], War Office, Whitehall, stating whether they wish to receive the insignia by post or at a County Investiture. Particulars of service by which applicants can be identified should be given.

